# A Closed-Loop Method for Multiperiod Intelligent Information Processing with Cost Constraints under the Fuzzy Environment

**DOI:** 10.1155/2022/3871129

**Published:** 2022-09-07

**Authors:** Ming Fu, Lifang Wang, Xueneng Cao, Bingyun Zheng, Xianxian Zhou, Shishu Yin

**Affiliations:** ^1^School of Management Science and Engineering, Anhui University of Finance & Economics, Bengbu 233030, Anhui, China; ^2^School of International Trade and Economics, Anhui University of Finance & Economics, Bengbu 233030, Anhui, China

## Abstract

From trivial matters in life to major scientific projects related to the fate of mankind, decision-making is everywhere. Whether high-quality decisions can be made often directly affects the development of affairs, especially when sudden disasters occur. As the basis of decision-making, data are crucial. The continuously probabilistic linguistic set, a data structure of the fuzzy mathematics, is selected in the paper to collect original data after careful comparisons, because this data structure can fully consider the hesitation of decision-makers and the fuzziness of complex problems. Although all alternatives are costly, the costs of different alternatives still vary greatly; obviously, the low-cost alternative is better than others when the same predetermined goal can be achieved, which is one of the research objectives and characteristics of this paper. Different from other researchers who only take the cost as one of the decision-making indicators, the algorithm proposed in the paper pays much more attention on the cost reduction. When dealing with an emergency, it is often difficult to solve the problem by taking measures only once; usually, multiple rounds of measures are needed. Each round of decision-making has both connections and differences, and the multiround decision-making model is proposed and built in the paper. Different from traditional linear structures, the model mainly adopts the closed-loop structure, which divides the whole process into multiple sub-decision-making points, the severities measured at the current time point will be compared with the values estimated at the latter time point, and then, the differences will be input into the system, the corresponding automatic adjustment modules will be activated immediately according to the values. The accuracy of the system can be verified and adjusted in time by the closed-loop control module. Finally, several experiments are carried out and the results show that the algorithm proposed in the paper is more effective and the cost is lower.

## 1. Introduction

People are always faced with all kinds of decision-making problems, how to make an appropriate decision in time is a scientific problem and has become one of research hotspots in the academic circle.

There are several different descriptions for the definition of decision-making. Simon believes that decision-making is essentially management [1]; Mikesell and Griffin, management professors, point out that decision-making is a process in which an appropriate alternative will be selected from multiple alternatives [2]; the American scholars Ebers and Maurer believe that decision-making should also include all activities, which must be carried out before making the final decision [3]. Generally speaking, decision-making is regarded as the process in which individuals or groups make appropriate decisions for specific goals.

Decision-making problems can be roughly divided into three categories from the perspective of known conditions: (1) the deterministic decision-making problem, such problems have clear alternatives and expected results; (2) the risky decision-making problem, the predetermined goal is clear; however, there are many paths to the goal, every path has certain risks and uncertainties, and fortunately, the probabilities can be roughly calculated; (3) the uncertain decision-making problem, it is similar to the risky decision-making problem; however, the probabilities can only be estimated, and even worse, there may be certain deviations in the estimated values. The problem studied in this paper belongs to the third category, which has many uncertainties and is the most complex of the three categories.

Information collection is a basic and key step of the decision-making; however, most information provided by interviewees is uncertain, vague, and hard to be denoted mathematically, how to scientifically record uncertain information is the first problem to be solved. In 1965, the Professor Zadeh has put forward the concept of the fuzzy set, which provided a new idea for solving such problems [4], the main contribution is that the concept of the membership degree has been proposed; subsequently, the theory has been widely recognized and developed rapidly, and various forms have been expanded, such as the interval-valued fuzzy set [5], the n-type fuzzy set [6], the intuitionistic fuzzy set [7], the interval intuitionistic fuzzy set [8], the hesitant fuzzy set [9], and the probabilistic linguistic set [10]. The main features of these fuzzy sets can be briefly summarized as follows: the membership degrees are described by interval values in the interval-valued fuzzy set; the membership degrees are represented by sets in the n-type fuzzy set; both the membership degree and the nonmembership degree can be considered in the intuitionistic fuzzy set; beyond that, the hesitation degrees, which are denoted by interval values, are included in the interval intuitionistic fuzzy set. The hesitations of the decision-makers can be described in the hesitant fuzzy set; in addition, the structure is concise and efficient, and therefore, the theory of the hesitant fuzzy set has become one of research hotspots in recent years. The probabilistic linguistic set is developed on the basis of the hesitant fuzzy set, and it adds occurrence probabilities to membership degrees, so as to increase further descriptions for membership degrees.

Mathematics is recognized as one of the best analytical tools. In order to use mathematical tools to carry out researches, scholars have put forward several basic mathematical concepts for fuzzy sets. Xia and Xu first gave the mathematical definition of the hesitant fuzzy set [11], and Liao and Xu defined some special hesitant fuzzy sets from the perspective of solving practical problems [12], such as the empty set *O*^*∗*^, the complete set *E*^*∗*^, and the meaningless set Θ^*∗*^. Unfortunately, fuzzy sets cannot be added, subtracted, multiplied, and divided directly; for this reason, several basic operation methods for fuzzy sets are proposed by scholars. Torra defined the complement, union, and intersection operations for hesitant fuzzy elements [13]. Xu and Xia conducted further researches and proposed the addition, multiplication, number multiplication, and power operations for hesitant fuzzy elements [14]; on this basis, Liao and Xu proposed the definitions of subtraction and division [15].

In addition, fuzzy elements cannot be compared directly like real numbers. Therefore, Xia and Xu have put forward the concept of the score value, which provides a method for comparing different fuzzy elements; however, when the score values are equal, it needs to be further judged with the help of the variance values [16], which was proposed by Liao et al.

Unfortunately, the basic operation methods mentioned above can only meet simple aggregation requirements and would be unable to finish the calculation when a large number of fuzzy elements participate. Therefore, researchers proposed several effective fuzzy aggregation operators. Xia and Xu proposed the hesitant fuzzy-weighted averaging (HFWA) operator and the hesitant fuzzy hybrid averaging (HFHA) operator in the paper listed in the Reference [11] mentioned above, considering the importance of location and data simultaneously. Liao and Xu defined a series of new hesitant fuzzy mixed integration operators and studied their boundaries and relationships [17]. Zhu and Xu proposed the hesitant fuzzy Bonferroni average operator and the weighted hesitant fuzzy Bonferroni average operator from the perspective of logical relationships, and studied their monotonicity, commutativity, and boundedness [18].

In particular, due to its outstanding structure, the theory of the probabilistic hesitation fuzzy set has been developing rapidly. Zhang et al. studied the preference relationships, ranking methods, basic operation rules, and aggregation operators [19]. Hao et al. studied the basic properties of the probabilistic dual hesitant fuzzy sets and proposed the entropy measurement methods, the comparison methods, and the aggregation operators [20], such as the weighted average operator and the geometric average operator. On this basis, Garg and Kaur studied the distance measurement methods of probabilistic dual hesitant fuzzy sets [21]. Ye proposed the correlation coefficients of probabilistic hesitant fuzzy sets in discrete and continuous cases, respectively [22]. Li and Wang proposed the concept of the probability hesitation fuzzy likelihood [23]. These theories have built a solid foundation for the probabilistic hesitation fuzzy theory.

Scholars have also conducted in-depth discussions on decision-making methods. The main idea can be simply summarized as using operators to aggregate estimation data and then rank alternatives according to the score values. These methods can be roughly divided into two categories: (1) optimize the aggregation operators and (2) innovate decision-making methods. For the first category, Jiang and Ma proposed the probability hesitation fuzzy frank-weighted average operator and the probability hesitation fuzzy frank-weighted geometric operator, and then discussed the relationships between them [24]. Zhao et al. considered the psychological preferences of decision-makers and proposed the probabilistic hesitant fuzzy Einstein aggregation operator [25]. Shao et al. proposed the probabilistic hesitation fuzzy priority integration operator after considering the internal correlations of indicators [26]. Li et al. proposed a new probabilistic hesitant fuzzy priority aggregation operator, which can make full use of the priority relationships among indicators [27]. For the second category, on the one hand, several commonly used methods in the field of the decision-making have been extended to the probabilistic hesitation fuzzy environment, such as the TOPSIS method, the QUALIFLEX method, and the LINMAP method; on the other hand, other theories or methods are introduced into the probabilistic hesitation fuzzy environment and make the theory more diversified. Zhou and Xu introduced several financial concepts into fuzzy sets and then applied the hybrid algorithm to the practice of the stock investment decision-making [28]. Tian et al. established a consensus process based on the probability hesitation fuzzy preference relationships and the prospect theory, and then applied it to financial venture investment [29]. Wu et al. introduced the GM (1,1) model of the grey theory and applied it to coal mine safety production [30]. Guo et al. introduced time series analysis and established a time series prediction model based on hesitation probability fuzzy sets [31]. For this article, we not only optimize the aggregation operators but also innovate decision-making methods; by comparison, the main work of the paper is to innovate the decision-making methods, and especially, the closed-loop control model is combined with the fuzzy decision-making algorithm.

## 2. The Basic Theories

This section will briefly introduce some important basic theories, which will be used in the following chapters, and it is helpful for other researchers to better understand the algorithm proposed in this paper.

### 2.1. The Continuously Probabilistic Linguistic Set

The continuously probabilistic linguistic set is an extended form of the probabilistic linguistic set, which overcomes the disadvantage of the limited number of possible values in the probabilistic linguistic set. The definition of the continuously probabilistic linguistic set (CPLS) can be mathematically described by the following equation:

(1) $$\begin{array}{c} L_{rs}=\left\{\gamma_{l}|p_{l}\gamma_{l}\in \left[0,1\right],p_{l}\in \left[0,1\right],l=1,2,\cdots ,m,\sum_{l=1}^{m}p_{l}=1\right\}. \end{array}$$

In the above definition, the evaluation value is recorded by the symbol *γ*_*l*_ and its corresponding probability is recorded by the symbol *p*_*l*_; the restraint condition *γ*_*l*_ ∈ [0,1] points out the range of evaluation values, and the greater the value of the *γ*_*l*_, the higher the evaluation acquired from experts; similarly, the restraint condition *p*_*l*_ ∈ [0,1] points out the range of probability values, and the greater the value of the *p*_*l*_, the greater the occurrence probability of the corresponding evaluation value; the pair of the symbol *γ*_*l*_*|p*_*l*_ can be called the continuously probabilistic linguistic element (CPLE); the restraint condition *l*=1,2, ⋯, *m* indicates the value range of the *l*, and the symbol *m* indicates the total number of evaluation values in the CPLS; the restraint condition ∑_*l*=1_^*m*^*p*_*l*_=1 indicates that the sum of all the probability values in any CPLS must equal to 1.

Unlike real numbers, CPLSs cannot be directly compared with each other, how to compare CPLSs is a difficult problem in front of researchers. The score function, which is first proposed by Farhadinia, can handle this problem effectively [32], and the calculation results are real numbers; therefore, they are easy to compare with each other. The definition of the score function can be mathematically described as equation (2). Generally, the score value of the CPLS represents the final evaluation result.

(2) $$\begin{array}{c} S\left(L_{rs}\right)=\sum_{l=1}^{m}\gamma_{l}\cdot p_{l}. \end{array}$$

It is also necessary to briefly introduce several other commonly used calculation formulas of CPLSs, which are listed as follows:

(3) $$\begin{array}{c} {\left(L_{rs}\right)}^{\lambda }=\underset{\gamma_{l}\in L_{rs}}{\cup }\left\{\gamma_{l}^{\lambda }|p_{l}l=1,2,\cdots ,m\right\},\\ \lambda L_{rs}=\underset{\gamma_{l}\in L_{rs}}{\cup }\left\{1-{\left(1-\gamma_{l}\right)}^{\lambda }|p_{l}l=1,2,\cdots ,m\right\},\\ L_{rs}⊕L_{pq}=\underset{\gamma_{l_{1}}\in L_{rs},\gamma_{l_{2}}\in L_{pq},p_{l_{1}}\in L_{rs},p_{l_{2}}\in L_{pq}}{\cup }\left\{\gamma_{l_{1}}+\gamma_{l_{2}}-\gamma_{l_{1}}\gamma_{l_{2}}|p_{l_{1}}p_{l_{2}}l_{1}=1,2\cdots ,m_{1}l_{2}=1,2\cdots ,m_{2}\right\},\\ L_{rs}⊗L_{pq}=\underset{\gamma_{l_{1}}\in L_{rs},\gamma_{l_{2}}\in L_{pq},p_{l_{1}}\in L_{rs},p_{2}\in L_{pq}}{\cup }\left\{\gamma_{l_{1}}\gamma_{l_{2}}|p_{l_{1}}p_{1_{2}}l_{1}=1,2\cdots ,m_{1}l_{2}=1,2\cdots ,m_{2}\right\}. \end{array}$$

We can find that only one CPLS is involved in the first and the second calculation formulas; while there are two CPLSs involved in the third and the fourth calculation formulas, more calculation formulas can be obtained according to these four basic formulas.

### 2.2. The Collaborative Decision-Making Problem

The definition of the collaborative decision-making can be simply described as a process in which several experts try to find the most appropriate alternative from multiple alternatives according to values of key indicators [33]. The experts can be denoted as *E*={*E*_1_, *E*_2_, ⋯, *E*_*m*_}, and the alternatives can be denoted as *A*={*A*_1_, *A*_2_, ⋯, *A*_*n*_} mathematically.

The emergency decision-making is an important branch of collaborative decision-making problems, and they have many similarities [34], while there are great differences in complexity between them. The main difference is that the emergency decision-making problem has strict restrictions on the time, and the information acquired by experts is limited; even worse, it is always difficult for experts to evaluate alternatives with single values, and they often hesitate among multiple values. Fortunately, the introduction of the continuously probabilistic linguistic set can handle this problem efficiently [35], and all the possible evaluation information for an alternative given by experts can be recorded, which avoids the loss of the original information.

A simple example is given to illustrate the above theory. Supposing dangerous chemicals suddenly leak on the highway and the emergency threatens the safety of people around and causes damage to the surrounding environment. Several experts are urgently summoned to find solutions for the incident, and then, they are asked to assess each solution within a limited time. It is assumed that there are three experts and four alternatives available to handle this incident, which can be denoted as *A*={*A*_1_, *A*_2_, *A*_3_, *A*_4_} and *E*={*E*_1_, *E*_2_, *E*_3_}, respectively. The CPLSs mentioned above can be used to record all the original evaluation information. Supposing the evaluation information given by the third expert for the second alternative is denoted as *L*_23_={0.3*|*0.4, 0.36*|*0.42, 0.38*|*0.18}, the values in the set {0.3, 0.36, 0.38} are evaluation values, and the values in the set {0.4, 0.42, 0.18} are the corresponding probability information, the calculation process of the score value is *S*_23_=0.3 × 0.4+0.36 × 0.42+0.38 × 0.18=0.3396.

The situation of emergencies always changes dynamically over time [36]; therefore, decisions need to be made according to the actual situations at different stages, and these problems will be discussed in detail in the next chapter of this paper.

### 2.3. The Information Aggregation Operators

The scattered information given by experts separately must be aggregated and obtained the final evaluation value for each alternative [37]. At present, there are several different aggregation methods [38], and the dynamic hesitant probability fuzzy weighted arithmetic (DHPFWA) operator is selected in this paper after comparisons because of its simple and intuitive characteristics.

Supposing a total of *k* experts have, respectively, given their evaluation information for the alternative *A*_*r*_, which can be denoted mathematically as *L*_*r*_={*L*_*r*1_, *L*_*r*2_, ⋯, *L*_*rk*_}, the weights of experts can be denoted as *ω*=(*ω*_1_, *ω*_2_, ⋯, *ω*_*k*_), which can be obtained according to their past experiences and authorities in this field; the greater the value is, the more important the evaluation information given by the expert is [39]; and the weights satisfy the constraints, which are *ω*_*i*_ ∈ (0,1) and ∑_*i*=1_^*k*^*ω*_*i*_=1. Equation (3) gives the specific calculation method of the DHPFWA operator.

(4) $$\begin{array}{c} DHPFWA\left(L_{r}\right)=\overset{k}{\underset{i=1}{⊕}}\left(\omega_{i}L_{ri}\right)=\underset{\gamma_{l_{1}}\in L_{r1},\gamma_{l_{2}}\in L_{r2}\cdots \gamma_{l_{k}}\in L_{rk}p_{l_{1}}\in L_{r1},p_{l_{2}}\in L_{r2}\cdots p_{l_{k}}\in L_{rk}}{\cup }\left\{1-\prod_{i=1}^{k}{\left(1-\gamma_{l_{i}}\right)}^{\omega_{i}}|p_{l_{1}}p_{l_{2}}\cdots p_{l_{k}}\right\}, \end{array}$$

where *l*_1_=1,2 ⋯ , *m*_1_, *l*_2_=1,2 ⋯ , *m*_2_, *l*_*k*_=1,2 ⋯ , *m*_*k*_, and we must point out that the values of *m*_1_, *m*_2_, ⋯, *m*_*k*_ are not necessarily equal to each other, which means that the total number of elements in different CPLSs can be completely unequal with each other. Let us give a simple example to illustrate the above theories, supposing the CPLSs *L*_*r*1_={034*|*036,038*|*035,040*|*029}, *L*_*r*2_={032*|*1} and *L*_*r*3_={035*|*07,039*|*03} are the evaluation information for the alternative *A*_*r*_ given by three experts, respectively. We can find that a total number of elements in the three CPLSs are *m*_1_=3, *m*_2_=1, and *m*_3_=2, respectively, and they are totally different from each other. Now further assume that the weights of the three experts are *ω*=(0.32, 0.27, 0.41), and the aggregated value of the three CPLSs can be calculated according to equation (3), which is shown as follows:

(5) $$\begin{array}{c} DHPFWA\left(L_{r}\right)=\overset{3}{\underset{i=1}{⊕}}\left(\omega_{i}L_{ri}\right)\\ =\left(\begin{array}{c} 0.338811|0.252,0.351907|0.245\\ 0.358672|0.203,0.355806|0.108\\ 0.368566|0.105,0.375157|0.087 \end{array}\right). \end{array}$$

We can find that the aggregated value is also in the form of CPLS and cannot be compared with other values directly [40], the score value can be further calculated according to equation (2) mentioned in Section 2.1, which is shown as follows:

(6) $$\begin{array}{c} S\left(L_{r}\right)=\sum_{l=1}^{m}\gamma_{l}\cdot p_{l}=0.354173. \end{array}$$

The form of the score value is very simple, and it is a real number, which is easy to be compared with other values and perform algebraic operations.

### 2.4. The Decision-Making Problem with Cost Constraints

Obviously, the cost is one of the most important constraints in the decision-making process, which cannot be ignored [41]. Although every alternative for dealing with emergencies is costly, while there are still wide gaps among different alternatives. The more rigorous the alternative is designed; usually, the better effect can be acquired, while the disadvantage is also obvious, which often have a great adverse impact on the local economy and increase burdens on the people and the government [42]. The costs include not only economic costs but also casualties, labour costs, environmental pollution, and expected income loss and so on; particularly, the casualties are the most important cost and must be seriously considered in the decision-making process [43].

Through the above analysis, we believe that the most appropriate alternative is not necessarily the one that just has the best effect, the cost and the effect must be considered comprehensively, which is more in line with the actual situation [44].

The main idea of dealing with the decision-making problem with cost constraints can be briefly described as follows: first, we reorder all the alternatives according to their costs, which can be denoted as *A*={*A*_1_, *A*_2_, ⋯, *A*_*n*_}; the estimated costs of these alternatives can be denoted as Δ*η*={Δ*η*_01_, Δ*η*_12_, ⋯ ,Δ*η*_*k*−1*k*_}, in which the symbol Δ*η*_*i*−1*i*_ indicates the estimated cost from the time point *t*_*i*−1_ to the time point *t*_*i*_; the estimated effects acquired by implementing these alternatives can be denoted as Δ*τ*={Δ*τ*_01_, Δ*τ*_12_, ⋯ ,Δ*τ*_*k*−1*k*_}, and similarly, the symbol Δ*τ*_*i*−1*i*_ indicates the estimated effect acquired from the time point *t*_*i*−1_ to the time point *t*_*i*_. We give the definition of the effect per cost (EPC), which can be described as *ψ*={*ψ*_*i*−1*i*_, *i*=1,2, ⋯, *k*}, *ψ*_*i*−1*i*_=Δ*τ*_*i*−1*i*_/Δ*η*_*i*−1*i*_. The definition of the EPC firstly proposed in the paper can consider the cost and effect comprehensively, and we believe that the most appropriate alternative in the current time point is the one that has the lowest EPC.

### 2.5. The Closed-Loop Control System

The closed-loop control system is a concept of the automatic control theory in the engineering technology. Its principle can be briefly described as follows: part or all of the output signals will be sent back to the input of the system, the differential signals between the original input signals and the feedback signals will be calculated, and then, they will be input into the system to automatically adjust relevant parameters [45], which is helpful to avoid the system from deviating from the predetermined goal.

We find that there are always differences between the values estimated at the previous time point and the values measured currently, the closed-loop control system provides a way to solve this problem, and we try to construct a closed-loop control system in the decision-making field [46]. Specifically speaking, we calculate the differences of the values estimated at the previous time point and the values measured currently and then input the differences into the decision-making system; thus, the relevant parameters of the system will be automatically adjusted in time according to the differences, and this is helpful to improve the evaluation accuracy of the system [47]. This is also one of the important improvements between the algorithm proposed in the paper and other decision-making methods.

## 3. The Closed-Loop Method of Collaborative Decision-Making

In this section, we will introduce the algorithm proposed in this paper in detail and build the mathematical model.

### 3.1. Mathematicize the Decision-Making Problem

Usually, it is impossible to achieve the expected goal by taking measures only once for dealing with emergencies, we need to adjust measures in time with the development of the situation. First of all, we make the following assumptions: the initial time point is denoted as *T*_0_, and the time point of achieving the expected goal is denoted as *T*_*k*_, and all time points are recorded in the set *T*={*T*_0_, *T*_1_, ⋯ , *T*_*k*_}. All the time intervals are recorded in the set Δ*T*={Δ*T*_01_, Δ*T*_12_, ⋯, Δ*T*_*k*−1*k*_}, and they can also be called periods. Generally, they are equal to each other, while, in some special cases, such as, when a major unexpected event occurs suddenly, a new time point must be inserted immediately.

The experts invited to deal with the emergency are denoted as *E*={*E*_1_, *E*_2_, ⋯, *E*_*m*_}, and their corresponding weights are denoted as *ω*={*ω*_1_, *ω*_2_, ⋯*ω*_*m*_}; the alternatives proposed by experts at the time point *T*_*i*_ are denoted as *A*_*i*_={*A*_*i*_^1^, *A*_*i*_^2^, ⋯, *A*_*i*_^*n*_*i*_^}; the values of the parameter *i*(*i*=0,1, ⋯*k*) indicate different time points; and the values of the *n*_*i*_(*i*=1,2, ⋯*k*) are not necessarily equal to each other. The experts will measure the current severity of the emergency according to the information acquired at each time point, these measurements will be denoted as *τ*={*τ*_0_, *τ*_1_, ⋯ ,*τ*_*k*_}, and each value *τ*_*i*_ in the set *τ* is in the form of CPLS.

### 3.2. The Subtraction between Any Two CPLSs

In order to build the feedback network, first of all, we need to calculate the differences between the estimated values made at the previous time point and the values measured at the current time point. Both data are in the form of CPLSs, and therefore, the subtraction between any two CPLSs must be required [48]; however, this theory is rarely mentioned by other researchers, and for this reason, the paper proposes a subtraction method between any two continuously probabilistic linguistic sets, which is shown as equation (4). We suppose the *L*_*rs*_ and the *L*_*pq*_ are two ordinary continuously probabilistic linguistic sets.

(7) $$\begin{array}{c} L_{d}=L_{rs}-L_{pq},\\ =\underset{\gamma_{l_{1}}\in L_{rs},\gamma_{l_{2}}\in L_{pq},p_{l_{1}}\in L_{rs},p_{1_{2}}\in L_{pq}}{\cup }\left\{\gamma_{l_{1}}-\gamma_{l_{2}}|p_{l_{1}}p_{l_{2}}l_{1}=1,2\cdots ,m_{1}l_{2}=1,2\cdots ,m_{2}\right\}. \end{array}$$

We find that the calculation result obtained by equation (4) is also a set, which can be called a special continuously probabilistic linguistic set. The main difference is that the values satisfy the constraint condition, which is −1 ≤ *γ*_*l*_1__ − *γ*_*l*_2__ ≤ 1 in the subtraction set, while the values satisfy the constraint condition, which is 0 ≤ *γ*_*l*_ ≤ 1 in any ordinary continuously probabilistic linguistic set. It can be further illustrated by a simple example, supposing that there are two ordinary CPLSs, which are recorded as *L*_*rs*_={04*|*02,041*|*08} and *L*_*pq*_={0.38*|*0.3, 0.41*|*0.1, 0.43*|*0.6}, respectively, and the subtraction result can be calculated according to equation (4) and the result is as follows:

(8) $$\begin{array}{c} L_{d}=L_{rs}-L_{pq}=\left\{0.02|0.06,-0.01|0.02,-0.03|0.12,0.03|0.24,0|0.08,-0.02|0.48\right\}. \end{array}$$

We can find that some values are greater than zero, while other values are less than zero, which is different from the definition of the ordinary continuously probabilistic linguistic set. The sum of probabilities is also equal to one, which is the same with the ordinary continuously probabilistic linguistic set. However, the above result is still not intuitive enough to reflect the differences; therefore, the score value of the special continuously probabilistic linguistic set needs to be further calculated. We must point out that the method mentioned in equation (2) is still applicable to the calculation of the special continuously probabilistic linguistic set, and the result is called as the special score value. The only difference is that the value range is 0 ≤ *S*(*L*) ≤ 1 for any ordinary CPLS, while the value range will be −1 ≤ *S*(*L*_*d*_) ≤ 1 for the special CPLS. For example, the special score value of the above example can be calculated according to equation (2), and the result is as follows:

(9) $$\begin{array}{c} S\left(L_{d}\right)=\sum_{l=1}^{m}\gamma_{l}\cdot p_{l}=-0.0096. \end{array}$$

When the score value is less than zero, it indicates that the value measured currently is better than the value estimated at the previous time point; when the score value is greater than zero, it indicates that the value measured currently is worse than the value estimated at the previous time point; when the score value is equal to zero, it indicates that the value measured currently is exactly equal to the value estimated at the previous time point; however, this ideal situation is almost impossible to happen.

### 3.3. The Method of Obtaining the Most Appropriate Alternative

Let us illustrate the algorithm proposed in the paper in the chronological order. At the initial time point *T*_0_, the current severity of the emergency measured by experts is denoted as *τ*_0_, the specific form can be denoted as *τ*_0_={*τ*_0_^1^, *τ*_0_^2^, ⋯, *τ*_0_^*m*^}, each value *τ*_0_^*i*^ given by the corresponding expert *E*_*i*_ is in the form of the continuously probabilistic linguistic set, and the specific form of the *τ*_0_ is further described in Table 1. The alternatives proposed at the time point *T*_0_ are denoted as *A*_0_={*A*_0_^1^, *A*_0_^2^, ⋯, *A*_0_^*n*_0_^}, and the estimated severities for the next time point *T*_1_ are denoted as *τ*_1_′={*τ*_1_^1/′^, *τ*_1_^1/′^, ⋯, *τ*_1_^*n*_0_/′^}. When using different alternatives, each value *τ*_1_^*i*/′^ in the set *τ*_1_′ is also a set, which can be denoted as *τ*_1_^*i*/′^={*τ*_10_^*i*/′^, *τ*_11_^*i*/′^, ⋯, *τ*_1*m*_^*i*/′^}; for example, the symbol *τ*_1*j*_^*i*′^ indicates the severity that the expert *E*_*j*_ estimated at the time point *T*_1_ by using the alternative *A*_*i*_, and the specific form of the *τ*_1_′ is further described in Table 2. We must point out that all the elements in Table 2 are also in the form of the continuously probabilistic linguistic set. Each value *τ*_1_^*i*/′^(*i*=1,2, ⋯, *n*_0_) consists of the elements in the corresponding row of Table 2. For the sake of simplicity, the specific forms of the elements in Table 2 are not given and they are similar to the elements in Table 1.

All the scattered information provided by experts can be aggregated by the *DHPFWA* operator, and then, the score value can be further calculated, and these theories have already been introduced in Section 2.3. Equations (5) and (6) are specific expansion forms for this problem. The calculation result of the DHPFWA(*τ*_0_) is in the form of the continuously probabilistic linguistic set, and the symbol *m* indicates the total number of elements in the DHPFWA(*τ*_0_).

(10) $$\begin{array}{c} \text{DHPFWA}\left(\tau_{0}\right)=\overset{m}{\underset{i=1}{⊕}}\left(\omega_{i}\tau_{0i}\right),=\underset{\gamma_{l_{1}}\in \tau_{01},\gamma_{l_{2}}\in \tau_{02}\cdots \gamma_{l_{k}}\in \tau_{0m}p_{l_{1}}\in \tau_{01},p_{l_{2}}\in \tau_{02}\cdots p_{l_{k}}\in \tau_{0m}}{\cup }\left\{1-\prod_{i=1}^{m}{\left(1-\gamma_{l_{i}}\right)}^{\omega_{i}}|p_{l_{1}}p_{l_{2}}\cdots p_{l_{k}}\right\}, \end{array}$$

(11) $$\begin{array}{c} S\left(T_{0}\right)=S\left(DHPFWA\left(\tau_{0}\right)\right)=\sum_{l=1}^{m^{\prime }}\gamma_{l}\cdot p_{l}. \end{array}$$

Similarly, the score values of the estimated severities at the time point *T*_1_ can also be calculated, which can be denoted as *S*′(*T*_1_)={*S*(*τ*_1_^1/′^), *S*(*τ*_1_^2/′^), ⋯, *S*(*τ*_1_^*n*_0_/′^)}; then, all the estimated effects can be calculated according to equation (7).

(12) $$\begin{array}{c} \Delta \tau_{01}=\left(\Delta \tau_{01}^{1/\prime },\Delta \tau_{01}^{2/\prime },\cdots ,\Delta \tau_{01}^{n_{0}/\prime }\right)\\ =S^{\prime }\left(T_{1}\right)-S\left(T_{0}\right)=\left\{S\left(\tau_{1}^{1/\prime }\right)-S\left(T_{0}\right),S\left(\tau_{1}^{2/\prime }\right)-S\left(T_{0}\right),\cdots ,S\left(\tau_{1}^{n_{0}/\prime }\right)-S\left(T_{0}\right)\right\}. \end{array}$$

Each value in the set Δ*τ*_01_ satisfies the constraint, which is −1 ≤ Δ*τ*_01_^*i*/′^ ≤ 1; when the value is negative, it indicates that the emergency has become worse after using the corresponding alternative *A*_*i*_; when the value is positive, it indicates that the emergency has been alleviated after using the corresponding alternative *A*_*i*_; and when the value is zero, it indicates that the emergency has not changed after using the corresponding alternative *A*_*i*_.

The cost of each period is recorded in the set Δ*η*={Δ*η*_01_, Δ*η*_12_, ⋯, Δ*η*_*k*−1*k*_}. The symbol Δ*η*_*ii*+1_ indicates the estimated cost from the time point *T*_*i*_ to the time point *T*_*i*+1_, because different alternatives *A*_*i*_={*A*_*i*_^1^, *A*_*i*_^2^, ⋯, *A*_*i*_^*n*_*i*_^} for dealing with the emergency will produce different costs, and the Δ*η*_*ii*+1_ is also a set, which can be denoted as Δ*η*_*ii*+1_={Δ*η*_*ii*+1_^1^, Δ*η*_*ii*+1_^2^, ⋯, Δ*η*_*ii*+1_^*n*_*i*_^}.

For the first period, the effect per cost *ψ*_01_ of using different alternatives can be calculated according to equation (8); obviously, the result is a set.

(13) $$\begin{array}{c} \psi_{01}=\left\{\psi_{01}^{i}|\psi_{01}^{i}=\frac{\Delta \tau_{01}^{i/\prime }}{\Delta \eta_{01}^{i}}i=1,2,\cdots ,n_{0}\right\}. \end{array}$$

The most appropriate alternative at this time point is the one that has the lowest EPC, which is shown as follows:

(14) $$\begin{array}{c} \psi_{01}^{j*}=\overset{n_{0}}{\underset{i=1}{\min\left(\psi_{01}^{i}\right)}}. \end{array}$$

Similarly, the most appropriate alternative at other time points can be obtained by this method.

### 3.4. The Construction of the Closed-Loop System

The most appropriate alternative *A*_*j*_ found in the previous step will be implemented immediately. The current severity of the emergency will be measured again at the time point *T*_1_, which can be denoted as *τ*_1_. The *τ*_1_ is a set that contains several values {*τ*_1_^1^, *τ*_1_^2^, ⋯, *τ*_1_^*m*^} given by different experts, respectively, according to the information acquired at the time point *T*_1_, and the specific form of the *τ*_1_ is further described in Table 3.

The differences between the values estimated at the initial time point *T*_0_ and the values measured at the first time point *T*_1_ will be calculated, and the calculation method is shown in equation (10), and its specific form is further described in Table 4.

(15) $$\begin{array}{c} d_{1}=\left\{d_{1}^{1},d_{1}^{2},\cdots ,d_{1}^{m}\right\}\\ =\left\{\tau_{11}^{j/\prime }-\tau_{1}^{1},\tau_{12}^{j/\prime }-\tau_{1}^{2},\cdots ,\tau_{1m}^{j/\prime }-\tau_{1}^{m}\right\}. \end{array}$$

We must point out that all the *τ*_1_^*i*^(*i*=1,2, ⋯, *m*) and the *τ*_1*i*_^*j*/′^(*i*=1,2, ⋯, *m*) are in the form of CPLS; therefore, each calculation equation *d*_1_^*i*^=*τ*_1*i*_^*j*/′^ − *τ*_1_^*i*^(*i*=1,2, ⋯, *m*) is a subtraction between CPLSs, and they must be calculated according to equation (4) mentioned in Section 3.2. All the differences *d*_1_^*i*^(*i*=1,2, ⋯, *m*) are also in the form of CPLS, and they will be aggregated according to equation (5) and equation (6) to obtain the total difference of the first period, which can be denoted as *S*(*d*_1_).

**Figure 1:**
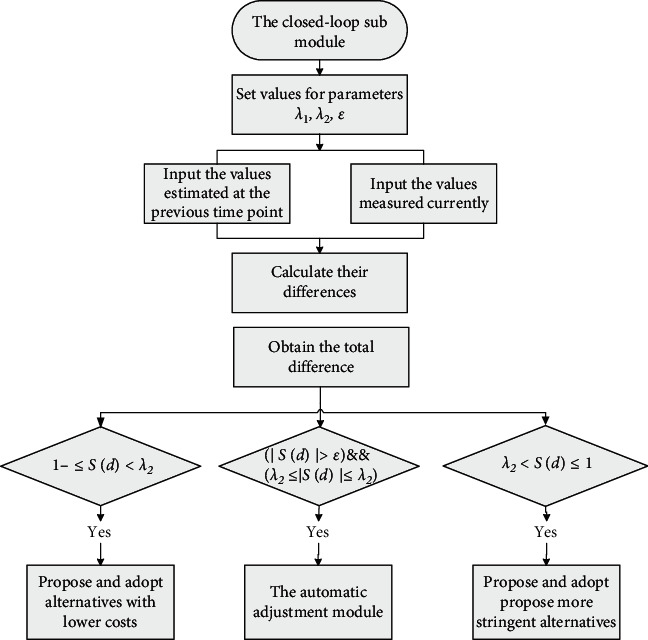
The flow chart of the closed-loop submodule.

The flow chart of the closed-loop submodule is shown in Figure 1. At this time, the system will enter the automatic adjustment stage. Four parameters that are denoted as *λ*_1_, *λ*_2_, *ε*, and *ς* will be set in advance, and the inequalities −1 ≤ *λ*_1_ ≤ −*ε* ≤ 0 ≤ *ε* ≤ *λ*_2_ ≤ 1 and 0 ≤ *ς* ≤ 1 hold. The smaller the value of *ε* is set, the higher the system accuracy is required; the larger the value of *λ*_1_ is set, the easier it is for the system to conduct conservative evaluation; the larger the value of *λ*_2_ is set, the easier it is for the system to conduct optimistic evaluation; and the greater the value of *ς* is set, the easier the predetermined goal can be achieved. If the inequality |*S*(*d*_1_)| ≤ *ε* holds, it indicates that the system works well and no adjustment is required; if the inequalities |*S*(*d*_1_)| > *ε* and *λ*_1_ ≤ *S*(*d*_1_) ≤ *λ*_2_ hold, it indicates that only minor adjustments are needed and the automatic adjustment method will be activated immediately; if the inequality *λ*_2_ < *S*(*d*_1_) ≤ 1 holds, it indicates that the system is too optimistic, experts are not fully aware of the severity and the development trend of the accident, and the system can be adjusted from two aspects: the first suggestion is that experts must propose more stringent alternatives, and the other suggestion is that experts should reduce the estimated values; if the inequality −1 ≤ *S*(*d*_1_) < *λ*_1_ holds, it indicates that the system is too pessimistic, the alternative used has achieved better results than expected. Similarly, the system can also be adjusted from two aspects: the first suggestion is that the experts can propose looser alternatives with lower costs, and the other suggestion is that the experts should appropriately raise the estimated values.

### 3.5. The Automatic Adjustment Algorithm

The symbol *ε* mentioned above is called the acceptable threshold. In this section, we propose an automatic adjustment algorithm for the estimated values and its specific steps are listed as follows:

**Step 1 .**

Appropriate values will be set for the system parameters *λ*_1_, *λ*_2_, and *ε* according to the actual situation of the emergency.

**Step 2 .**

Calculate the total differences of the current period *S*(*d*)={*S*(*d*_*i*_)*|i*=1,2, ⋯, *k*} by using the method mentioned in Section 3.4.

**Step 3 .**

Let us take the first period as an example to illustrate the algorithm, suppose the inequality *λ*_1_ ≤ *S*(*d*_1_) ≤ *λ*_2_ holds, and the inequality |*S*(*d*_1_)| ≤ *ε* does not hold.

**Step 4 .**

It can be divided into two categories according to the value of the *S*(*d*_1_). When the inequality *λ*_1_ ≤ *S*(*d*_1_) < −*ε* holds, first of all, the maximum value must be found from all the estimated values, supposing the symbol *γ*_*i*_ represents the maximum value, then increase *m* × |*S*(*d*_1_)| to the value, and the symbol *m* represents the total number of experts. On the other hand, when the inequality *ε* < *S*(*d*_1_) ≤ *λ*_2_ holds, similarly, the maximum value should decrease *m* × *S*(*d*_1_). We can summarize that the adjustment method can be unified for both categories after the above analysis, which can be shown as follows:

(16) $$\begin{array}{c} \gamma_{i}=\gamma_{i}-m\times S\left(d_{1}\right). \end{array}$$

**Step 5 .**

Similarly, the total difference *S*′(*d*_1_) can be calculated again according to the updated estimated values, and the step 3 and the step 4 will be repeated until the inequality |*S*(*d*_1_)| ≤ *ε* holds.

**Step 6 .**

The qualified estimated values will be obtained after several rounds of automatic adjustments.

**Figure 2:**
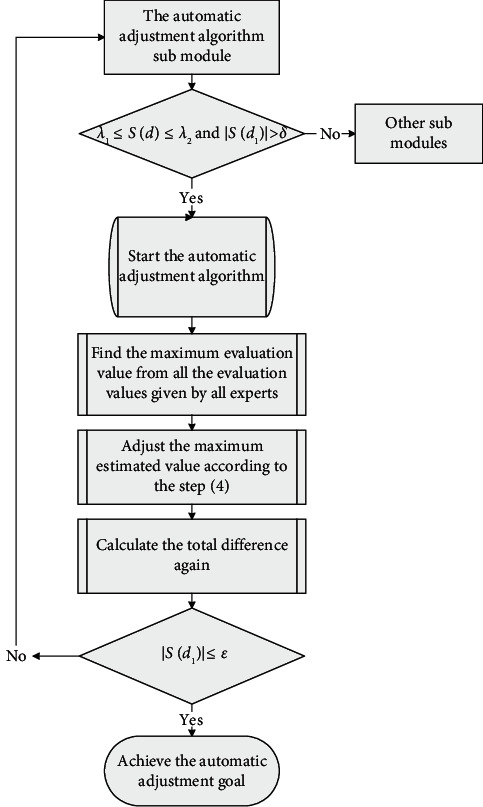
The flow chart of the automatic adjustment submodule.

The automatic adjustment algorithm has two advantages: the first advantage is that the algorithm is efficient and highly automated, and another advantage is that the original estimated information given by experts is minimally modified compared with other algorithms. The flow chart of the automatic adjustment submodule is shown in Figure 2.

### 3.6. The Brief Summary of the Algorithm Proposed in the Paper

**Figure 3:**
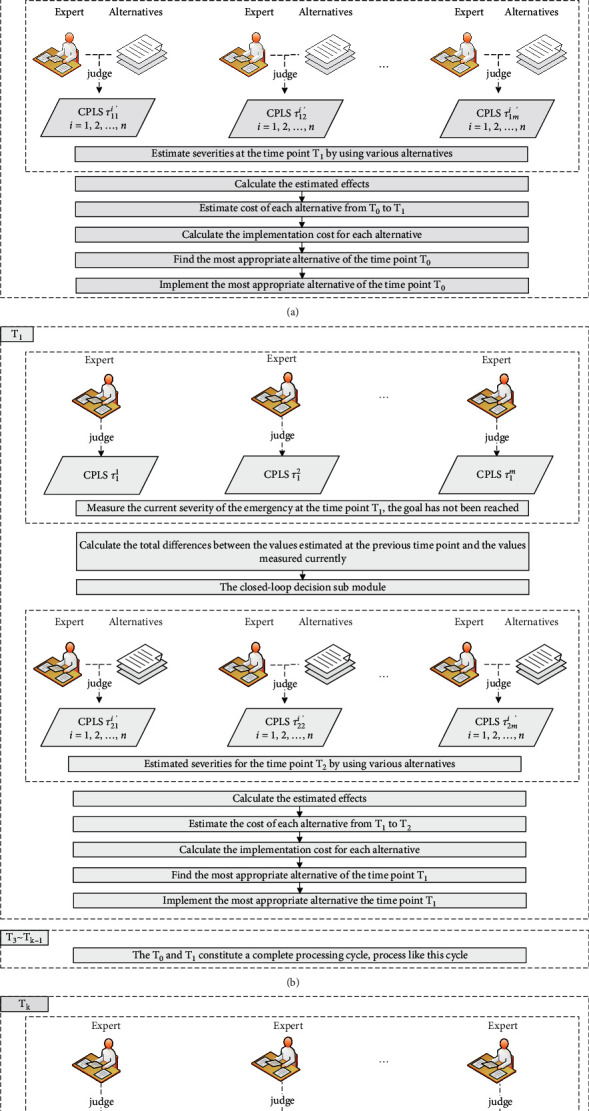
The overall flow chart of the algorithm proposed in the paper.

The overall flow chart of the algorithm proposed in the paper is shown in Figure 3. The whole algorithm is divided into multiple time points, which are denoted as {T_0_,T_1_, ⋯ ,T_*k*_}, and the time that spans any two adjacent time points can be called a period, such as ΔT_0_=[T_0_, T_1_].

At the time point T_0_, the current severity of the emergency will be measured by experts, and the data can be called measured values for short; then, the algorithm will judge whether the predetermined goal has been achieved or not according to the measured values. If the goal has been achieved, the algorithm will be terminated immediately; if the goal has not been achieved, experts will estimate the severities at the next time point when using different alternatives and the data obtained can be called estimated values for short. The estimated effects of different alternatives can be calculated according to the measured values and the evaluated values, and the cost of each alternative can be estimated according to specific measures. After above preparation, the effect per cost of each alternative can be calculated. Finally, the most appropriate alternative that has the lowest EPC will be found and it will be implemented immediately.

Similarly, at the time point T_1_, experts will measure the current severity of the emergency, and then, they will judge whether the predetermined goal has been achieved or not again. If the goal has been achieved, the algorithm will be terminated; if the goal has not been achieved, the total differences between the values estimated at the previous time point and the values measured currently will be calculated, and the corresponding automatic adjustment submodules will be activated according to the differences. The following processing methods are similar to the above steps, and the most appropriate alternative of this time point will be found and implemented.

From the time point *T*_2_ to the time point *T*_*k*−1_, the algorithm will repeat the above processes and the severity of the emergency will gradually decrease. The emergency will be effectively controlled after several rounds of treatment.

At the time point *T*_*k*_, experts will measure the current severity of the emergency, and they find that the inequality |1 − S(T_*k*_)| ≤ *ς* holds, which indicates that the predetermined goal has been achieved, the algorithm will be terminated immediately. The parameter *ς* is called the completion threshold. The emergency has been handled effectively with the lowest cost.

## 4. A Case of the Closed-Loop Collaborative Decision-Making Algorithm

### 4.1. The General Description of the Emergency

The whole world is facing the severe challenge of the COVID-19 (corona virus disease 2019), and the latest prediction shows that the epidemic will lead to a global economic recession and large-scale unemployment. It has caused a large number of infections; even worse, various prevention and control methods are not mature enough to fundamentally eradicate the infectious disease.

At present, the COVID-19 has been basically controlled in China; however, we found that the epidemic is still breaking out occasionally in some areas of China and has the trend of further expansion, and it has added great resistance to the employment and the economic development of China. The Chinese government has taken various measures to deal with the epidemic for many years; however, the epidemic situation is changing continuously over time. Obviously, this problem belongs to the dynamic decision-making problem; in addition, we can hardly hope to solve this problem through only a round of measures, and therefore, the multiround decision-making algorithm discussed in the paper is suitable to deal with this problem. The specific steps of the proposed algorithm will be introduced in this section according to the chronological order.

### 4.2. The Processing Methods at the Time Point *T*_0_

Let us take one of universities in the high-risk areas as an example to illustrate the algorithm and the university is facing the threat of the epidemic. The appropriate alternatives must be found out at different time points to prevent and control the epidemic. Supposing that a total of three experts are summoned to deal with this emergency, and they have put forward four response alternatives, which can be denoted as *A*_0_={*A*_0_^1^, *A*_0_^2^, *A*_0_^3^, *A*_0_^4^} at the initial time point *T*_0_. The predetermined goal is to minimize the adverse impact of the COVID-19 on normal teaching and student activities.

Table 5 lists the alternatives proposed by experts for handling the emergency at the initial time point (*T*_0_). We can find that the measures in the table have gradually become more and more stringent from top to bottom, and we must admit that the latter alternative is indeed better than the former alternative in controlling the epidemic situation; however, the disadvantage is that the cost will be higher; once again, we point out that the most appropriate alternative is not necessarily the most stringent alternative.

The current severities of the emergency measured by experts separately according to the available information are listed in Table 6, and the weights of experts are also given. Obviously, the predetermined goal has not been achieved. The scattered information can be aggregated according to equations (5) and (6). The score value obtained ranges from 0 to 1, and the symbol “0” indicates that the situation is extremely bad, while the symbol “1” indicates that the situation is perfect. The current severity is 0.1554, and the specific calculation processes are shown as follows:

(17) $$\begin{array}{c} DHPFWA\left(t_{0}\right)=\overset{3}{\underset{k=1}{⊕}}\left(\omega_{k}\tau_{0}^{k}\right)\\ =\left\{\begin{array}{c} 0.117281|0.096,0.136686|0.064,0.144651|0.16,0.130477|0.024\\ 0.149592|0.016,0.157438|0.04,0.147927|0.144,0.166659|0.096\\ 0.174347|0.24,0.160665|0.036,0.179116|0.024,0.18669|0.06 \end{array}\right\}, \end{array}$$

(18) $$\begin{array}{c} S\left(T_{0}\right)=\sum_{l=1}^{m}\gamma_{l}\cdot p_{l}\\ =0.1554. \end{array}$$

The values of the estimated severities at the time point *T*_1_ when using different alternatives are listed in Table 7. Similarly, the score values are calculated and their specific calculation steps are shown as follows:

(19) $$\begin{array}{c} \tau_{1}^{1/\prime }=\overset{3}{\underset{k=1}{⊕}}\left(\omega_{k}\tau_{1k}^{1/\prime }\right)=\left\{\begin{array}{c} 0.219122|0.144,0.225504|0.072,0.228737|0.024,0.238284|0.096\\ 0.244509|0.048,0.247663|0.016,0.225345|0.216,0.231675|0.108\\ 0.234882|0.036,0.244354|0.144,0.250529|0.072,0.253658|0.024 \end{array}\right\},\\ S\left(\tau_{1}^{1/\prime }\right)=\sum_{l=1}^{m}\gamma_{l}\cdot p_{l}=0.2333,\\ \tau_{1}^{2/\prime }=\overset{3}{\underset{k=1}{⊕}}\left(\omega_{k}\tau_{1k}^{2/\prime }\right)=\left\{\begin{array}{c} 0.287015|0.045,0.293015|0.075,0.29606|0.03,0.298616|0.105\\ 0.304519|0.175,0.307514|0.07,0.293504|0.045,0.29945|0.075\\ 0.302467|0.03,0.305|0.105,0.310849|0.175,0.313817|0.07 \end{array}\right\},\\ S\left(\tau_{1}^{2/\prime }\right)=\sum_{l=1}^{m}\gamma_{l}\cdot p_{l}=0.3031,\\ \tau_{1}^{3/\prime }=\overset{3}{\underset{k=1}{⊕}}\left(\omega_{k}\tau_{1k}^{3/\prime }\right)=\left\{\begin{array}{c} 0.345523|0.09,0.352035|0.045,0.35869|0.015,0.356817|0.09\\ 0.363217|0.045,0.369757|0.015,0.351727|0.21,0.358178|0.105\\ 0.364769|0.035,0.362914|0.21,0.369253|0.105,0.375731|0.035 \end{array}\right\},\\ S\left(\tau_{1}^{3/\prime }\right)=\sum_{l=1}^{m}\gamma_{l}\cdot p_{l}=0.3587,\\ \tau_{1}^{4/\prime }=\overset{3}{\underset{k=1}{⊕}}\left(\omega_{k}\tau_{1k}^{4/\prime }\right)=\left\{\begin{array}{c} 0.373669|0.054,0.376717|0.036,0.3798|0.09,0.385363|0.036\\ 0.388354|0.024,0.391379|0.06,0.386471|0.126,0.389457|0.084\\ 0.392477|0.21,0.397926|0.084,0.400856|0.056,0.40382|0.14 \end{array}\right\},\\ S\left(\tau_{1}^{4/\prime }\right)=\sum_{l=1}^{m}\gamma_{l}\cdot p_{l}=0.3908. \end{array}$$

The score values of the estimated severities at the time point *T*_1_ can be recorded as *S*′(*T*_1_)={0.2333, 0.3031, 0.3587, 0.3908}. Subsequently, the estimated effects at the period Δ*T*_01_ can be calculated according to equation (7), which are shown as follows:

(20) $$\begin{array}{c} \Delta \tau_{01}=\left\{\Delta \tau_{01}^{1\prime },\Delta \tau_{01}^{2\prime },\Delta \tau_{01}^{3\prime },\Delta \tau_{01}^{4\prime }\right\}=\left\{0.0779,0.1477,0.2033,0.2354\right\}. \end{array}$$

The costs at the period Δ*T*_01_ can be denoted as Δ*η*_01_={Δ*η*_01_^1^, Δ*η*_01_^2^, Δ*η*_01_^3^, Δ*η*_01_^4^}when using different alternatives. Supposing the cost of the alternative *A*_1_ is normalized and is regarded as “1,” other values will be standardized based on this value. The estimated costs of all alternatives are Δ*η*_01_=(1,1.2, 1.7, 2).

Obviously, the alternative *A*_4_ has the best effect; however, its cost is also the highest, and therefore, the most appropriate alternative cannot be determined directly, and the effects per cost of all alternatives need to be further calculated according to equation (8), which are shown as follows:

(21) $$\begin{array}{c} \psi_{01}=|\left(\psi_{01}^{i}\psi_{01}^{i}=\frac{\Delta \tau_{01}^{i/\prime }}{\Delta \eta_{01}^{i}}i=1,2,3,4\right)\\ =\left\{0.0779,0.123083,0.119588,0.1177\right\}. \end{array}$$

The order of the alternatives can be denoted as *ψ*_01_^2^ > *ψ*_01_^3^ > *ψ*_01_^4^ > *ψ*_01_^1^ according to the values of EPCs; therefore, the alternative *A*_2_ is the most appropriate alternative at the time point *T*_0_, and it will be implemented immediately.

### 4.3. The Processing Methods at the Time Point *T*_1_

Similarly, the experts will measure the severities again at the time point *T*_1_ and their values are listed in Table 8.

Obviously, the predetermined goal has still not been achieved. In order to test and improve the accuracy of the system, the differences between the values estimated at the *T*_0_ and the values measured at the *T*_1_ will be calculated and their values are listed in Table 9.

(22) $$\begin{array}{c} d_{1}=\overset{3}{\underset{k=1}{⊕}}\left(\omega_{k}d_{1}^{k}\right)=\left\{\begin{array}{c} -0.004481|0.0054,-0.010407|0.0126,0.001529|0.0090,-0.004481|0.0210\\ 0.004565|0.0036,-0.001487|0.0084,-0.017554|0.0081,-0.023557|0.0189\\ -0.011467|0.0135,-0.017554|0.0315,-0.008391|0.0054,-0.014521|0.0126\\ 0.002194|0.0054,-0.003693|0.0126,0.008163|0.0090,0.002194|0.0210\\ 0.011179|0.0036,0.005168|0.0084,-0.011062|0.0081,-0.017027|0.0189\\ -0.005014|0.0135,-0.011062|0.0315,-0.001957|0.0054,-0.008048|0.0126\\ 0.006739|0.0126,0.000879|0.0294,0.012681|0.0210,0.006739|0.0490\\ 0.015683|0.0084,0.009700|0.0196,-0.006188|0.0189,-0.012125|0.0441\\ -0.000169|0.0315,-0.006188|0.0735,0.002873|0.0126,-0.003189|0.0294\\ 0.013339|0.0126,0.007518|0.0294,0.019241|0.0210,0.013339|0.0490\\ 0.022224|0.0084,0.016280|0.0196,0.000231|0.0189,-0.005668|0.0441\\ 0.006212|0.0315,0.000231|0.0735,0.009234|0.0126,0.003211|0.0294 \end{array}\right\},\\ S\left(d_{1}\right)=\sum_{l=1}^{m}\gamma_{l}\cdot p_{l}=-0.0005360609, \end{array}$$

The system parameters are set as *λ*_1_=−0.001, *λ*_2_=0.001, *ε*=00005, and *ς*=0.04. We can find that the inequality *λ*_1_ < *S*(*d*_1_) < *λ*_2_ holds; therefore, major adjustments are not required, and however, the inequality −*ε* ≤ *S*(*d*_1_) ≤ *ε* does not hold, which indicates minor adjustments are still required and the automatic adjustment module will be activated immediately. According to the algorithm, the maximum estimated value of the alternative *A*_0_^2^ in Table 7 can be found, the value 0.32 will increase to 0.3216081827 according to equation (11), and other values remain unchanged. The updated severities are shown in Table 10.

The total difference will be calculated again according to the data in Table 11, and the specific steps are shown as follows:

(23) $$\begin{array}{c} d_{1}^{\prime }=\overset{3}{\underset{k=1}{⊕}}\left(\omega_{k}d_{1}^{\prime /k}\right)=\left\{\begin{array}{c} -0.004481|0.0054,-0.010407|0.0126,0.001529|0.0090,-0.004481|0.0210\\ 0.004565|0.0036,-0.001487|0.0084,-0.017554|0.0081,-0.023557|0.0189\\ -0.011467|0.0135,-0.017554|0.0315,-0.008391|0.0054,-0.014521|0.0126\\ 0.002194|0.0054,-0.003693|0.0126,0.008163|0.0090,0.002194|0.0210\\ 0.011179|0.0036,0.005168|0.0084,-0.011062|0.0081,-0.017027|0.0189\\ -0.005014|0.0135,-0.011062|0.0315,-0.001957|0.0054,-0.008048|0.0126\\ 0.007346|0.0126,0.001490|0.0294,0.013285|0.0210,0.007346|0.0490\\ 0.016285|0.0084,0.010305|0.0196,-0.005573|0.0189,-0.011506|0.0441\\ -0.000442|0.0315,-0.005573|0.0735,0.003482|0.0126,-0.002576|0.0294\\ 0.013942|0.0126,0.008125|0.0294,0.019841|0.0210,0.013942|0.0490\\ 0.022822|0.0084,0.016881|0.0196,0.000842|0.0189,-0.005053|0.0441\\ 0.006819|0.0315,0.000842|0.0735,0.009840|0.0126,0.003820|0.0294 \end{array}\right\},\\ S^{\prime }\left(d_{1}\right)=\sum_{l=1}^{m}\gamma_{l}\cdot p_{l}=-\text{0}0001092876. \end{array}$$

We can find that the inequality −*ε* < *S*′(*d*_1_) < *ε* holds at this time, which indicates that the automatic adjustment module works well. The updated values in Table 10 can provide references for experts in the next estimation.

Since the inequality *λ*_1_ < *S*(*d*_1_) < *λ*_2_ holds, the most appropriate alternative at this time point is the same as the one at the previous time point; therefore, the alternative *A*_2_ is still the most appropriate alternative at the time point *T*_1_ and it will be implemented immediately.  Table 12 lists the estimated severities at the time point *T*_2_ when using different alternatives. Since all the alternatives proposed by experts have not changed, the costs remain unchanged.

### 4.4. The Processing Methods at the Time Point *T*_2_

In the same way, the experts will measure the severities again at the time point *T*_2_ and their values are listed in Table 13.

Obviously, the predetermined goal has not been achieved. The differences between the values estimated at the time point *T*_1_ and the values measured at the time point *T*_2_ will be calculated, which are shown in Table 14. The total difference will be aggregated according to the data in Table 14.

(24) $$\begin{array}{c} d_{2}=\overset{3}{\underset{k=1}{⊕}}\left(\omega_{k}d_{1}^{k}\right)=\left\{\begin{array}{c} -0.17952|0.0105,-0.18553|0.0063,-0.19147|0.0042,-0.16727|0.0420\\ -0.17343|0.0252,-0.17952|0.0168,-0.18265|0.0245,-0.18868|0.0147\\ -0.19464|0.0098,-0.17038|0.0980,-0.17655|0.0588,-0.18265|0.0392\\ -0.16356|0.0105,-0.16949|0.0063,-0.17536|0.0042,-0.15148|0.0420\\ -0.15756|0.0252,-0.16356|0.0168,-0.16679|0.0245,-0.17274|0.0147\\ -0.17862|0.0098,-0.15468|0.0980,-0.16077|0.0588,-0.16679|0.0392\\ -0.17181|0.0045,-0.17779|0.0027,-0.18369|0.0018,-0.15965|0.0180\\ -0.16577|0.0108,-0.17181|0.0072,-0.17493|0.0105,-0.18092|0.0063\\ -0.18684|0.0042,-0.16273|0.0420,-0.16887|0.0252,-0.17493|0.0168\\ -0.15596|0.0045,-0.16186|0.0027,-0.16768|0.0018,-0.14396|0.0180\\ -0.15000|0.0108,-0.15596|0.0072,-0.15917|0.0105,-0.16508|0.0063\\ -0.17092|0.0042,-0.14714|0.0420,-0.15319|0.0252,-0.15917|0.0168 \end{array}\right\},\\ S\left(d_{2}\right)=\sum_{l=1}^{m}\gamma_{l}\cdot p_{l}=-\text{0}1659861303. \end{array}$$

We can find that the inequality *S*(*d*_2_) < *λ*_1_ holds, which indicates that the actual effects of the alternative are much better than the estimated effects and major adjustments must be required. Experts need to check the system carefully to find out whether any important information for decision-making is missing. The alternative with lower cost should be adopted, if the alternative adopted in the last round of decision-making is already the cheapest alternative, experts should propose a new and cheaper alternative. Since the inequality Δ*η*_23_^1^ < Δ*η*_23_^2^ holds in this case, which indicates that the alternative with lower cost exists, therefore, there is no need to propose a new alternative, and the alternative *A*_1_ will be the most appropriate alternative at the time point *T*_2_, and it will be implemented immediately.

Duo to the good effect of the alternative, the experts will give more optimistic estimated values in the next round of estimations, which are shown in Table 15.

### 4.5. Achieve the Predetermined Goal

The experts will measure the severities of the emergency again at the time point *T*_3_, and their values are listed in Table 16, and then, the score value will be calculated.

(25) $$\begin{array}{c} S\left(T_{3}\right)=\sum_{l=1}^{m}\gamma_{l}\cdot p_{l}=\text{0}968163. \end{array}$$

We can find that the inequality |1 − S(T_3_)| ≤ *ς* holds, which indicates that the emergency has almost been eliminated, and only routine inspections are required and the algorithm will be terminated.

## 5. The Comparisons and Discussions

Many scholars have also proposed several outstanding algorithms in the field of decision-making from various perspectives, and these algorithms have their characteristics and suitable application scopes [49]. The comparisons between the algorithms proposed in the paper and others will be made in this section, which will be helpful for finding out the advantages and disadvantages of the algorithm proposed in this paper.

### 5.1. The Hesitant Fuzzy Set and Its Processing Methods

The hesitant fuzzy set, a classic data structure, is one of the important definitions in the fuzzy mathematics [50], and its information aggregation operators and comparison methods are also quite mature; particularly, many complex data structures are developed from it. Unfortunately, the probability information of the evaluation values cannot be recorded together in the hesitant fuzzy set.  Table 17 lists the conversion values of Table 7 when the data are recorded in the form of hesitant fuzzy sets.

We find that only the evaluation values can be recorded, and all the corresponding probability information is missing. From the other point of view, it can be considered that all the probability values are equal to each other in any hesitant fuzzy set. Therefore, the hesitant fuzzy set is a special case of the continuously probabilistic linguistic set, and the continuously probabilistic linguistic set can record more detail information, which will make the algorithm more accurate fundamentally.

### 5.2. The Probabilistic Linguistic Set and Its Processing Methods

The probabilistic linguistic set (PLS) is also one of the efficient data structures, and it is widely used in the field of dealing with fuzzy problems, especially the collection and storage of the fuzzy data [51].

The total number of the possible evaluation values in the PLS is limited [52], and all the possible evaluation values are contained in the additive linguistic term set, which are denoted as *S*={*s*_*α*_*|α*=0,1, ⋯, 2*τ*}, and the symbol *τ* indicates a positive integer. The definition of the probabilistic linguistic set can be described mathematically as follows:

(26) $$\begin{array}{c} L_{p}=\left\{L_{l}\left(p_{l}\right)|L_{l}\in S,p_{l}\geq 0,l=1,2,\cdots ,m,\sum_{l=1}^{m}p_{l}=1\right\}. \end{array}$$

Obviously, the data structure CPLS proposed in the paper is developed from the probabilistic linguistic set and it not only inherits the advantages of the PLS but also overcomes its disadvantages, and it expands the number of possible evaluation values from limited to countless.

For the case mentioned above, the additive linguistic term set can be set as *S*={*s*_*α*_*|α*=0,1,2,3,4}, the symbol *s*_0_ indicates “terrible”; the symbol *s*_1_ indicates “bad”; the symbol *s*_2_ indicates “moderate”; the symbol *s*_3_ indicates “good”; and the symbol *s*_4_ indicates “perfect.” Let us also take the data in Table 7 as an example to illustrate the data structure, and the estimated values cannot be directly converted to the additive linguistic term sets; therefore, first, we should establish the transformation rules, which can be described as follows: the values will be set as *s*_0_ if the inequality 0 ≤ *τ*^1/′^ < 0.2 holds; the values will be set as *s*_1_ if the inequality 0.2 ≤ *τ*^1/′^ < 0.4 holds; the values will be set as *s*_2_ if the inequality 0.4 ≤ *τ*^1/′^ < 0.6 holds; the values will be set as *s*_3_ if the inequality 0.6 ≤ *τ*^1^ ′ < 0.8 holds; and the values will be set as *s*_4_ if the inequality 0.8 ≤ *τ*^1/′^ ≤ 1 holds.  Table 18 lists the transformed values when the data are recorded in the form of the probabilistic hesitant fuzzy sets.

We find that the values in the *A*_1_^1^, *A*_1_^2^, *A*_1_^3^ are equal to each other and all the evaluation values given by different experts are *s*_1_ and *s*_2_; obviously, the discrimination ability of this method is poorer than the algorithm proposed in the paper.

### 5.3. The Decision-Making Algorithms without the Cost Limitation

The cost limitation in the decision-making process is one of the characteristics of the algorithm proposed in the paper. Although many other algorithms have considered costs, they only take the cost as one of decision-making indicators and do not list it separately [53]. In some cases, we found that the increase in cost does not improve any effect. For the case discussed in the paper, the most appropriate alternatives will be *A*_4_ ~ *A*_4_ ~ *A*_4_ if only the effects are considered, the total cost will be *η*=Δ*η*_01_^4^+Δ*η*_12_^4^+Δ*η*_23_^4^=6. The final result is *A*_2_ ~ *A*_2_ ~ *A*_1_, which is obtained by the algorithm proposed in the paper, and the total cost is *η*′=Δ*η*_01_^2^+Δ*η*_12_^2^+Δ*η*_23_^1^=3.4. We can find that the same goal has been achieved, but the cost is saved by 43.3%, which verifies the superiority of the algorithm proposed in the paper from the perspective of the cost.

### 5.4. The Open-Loop Decision-Making Algorithms

At present, most decision-making algorithms adopt the open-loop mode; in other words, they fail to establish a set of feedback mechanisms [54]. Now we will demonstrate the method without feedback mechanisms to solve the above case and point out the differences between the method and the algorithm proposed in the paper.

The alternative *A*_2_ will still be the most appropriate alternative at the time point *T*_0_. The estimated values cannot be compared with the measured values at the time point *T*_1_; therefore, the accuracy of the system cannot be verified, the automatic adjustment module proposed in the paper cannot be activated, the system cannot be adjusted in time, and the error rate will be higher and higher with the increasing of time. One of the noticed differences will occur at the time point *T*_2_, the *A*_2_ instead of the *A*_1_ will be the most appropriate alternative if the feedback mechanism fails work, and the conclusion that only the alternative with lower cost is needed and the estimated values must be improved in the next estimation cannot be drawn, and this will directly lead to the increase of costs and processing cycles.

In short, the feedback mechanism is effective for timely verifying the correctness of the system, and it can save the total cost and reduce time effectively [55], which verifies the superiority of the algorithm proposed in the paper from the perspective of the accuracy.

## 6. Conclusions

When faced with emergencies, especially disasters, it is crucial to make timely and appropriate decisions; however, it is not easy to achieve this goal because of the limited time for making decisions and the fuzzy information that can be acquired.

The accuracy of data can directly affect the quality of the final decision, while we find that it is hard to record data accurately and scientifically. How to improve the accuracy of the collected data is the first problem to be solved. The data structure, the continuously probabilistic linguistic set, is adopted to save original data after comparisons. This data structure allows multiple possible values can be stored together in a record; meanwhile, the probability information of each possible value can also be stored together, and these characteristics can overcome the uncertainty and fuzziness in the process of data acquisition, which can improve the data quality to the greatest extent and lay a solid foundation for the later decision-making.

At present, most decision-making models adopt the linear structure and single-round mode, although these models have been elaborately designed, an important defect cannot be ignored; that is, it is impossible to verify the accuracy of the estimated results given by the system in time. In order to solve this problem, a new structure is proposed in the paper. The whole decision-making process is divided into multiple sub-decision-making stages, and each estimated result can be verified at the next decision-making time point. The estimated values and the current measured values are two different types of signals used in the system, the differences of the values estimated at the previous time point, and the values measured currently will be calculated by the fuzzy subtraction proposed in the paper. In general, there are certain differences between them, and the greater the difference, the lower the accuracy of the system. Due to time constraints, it is almost impossible for experts to reevaluate alternatives; fortunately, the paper proposes an automatic repair algorithm, which can solve this problem. The repair algorithm contains several submodules according to different situations, when the inequality *S*(*d*) ≤ |*ε*| holds, which indicates that the system works well and does not need any adjustment; when the inequalities *λ*_1_ ≤ *S*(*d*) < −*ε* or *ε* < *S*(*d*) ≤ *λ*_2_ hold, which indicates that the system needs minor adjustments and the automatic adjustment algorithm will be activated immediately; when the inequality *λ*_2_ < *S*(*d*) ≤ 1 holds, which indicates that the system is too optimistic and the actual situation is more serious than estimated; and when the inequality −1 ≤ *S*(*d*) < *λ*_1_ holds, which indicates that the system is too prudent and the actual effect is much better than estimated. The closed-loop decision-making system can be constructed through the establishment of the feedback mechanisms, and the accuracy of the whole model will be improved effectively.

The cost is one of the most important factors in the decision-making process, and we must point out again that the cost mentioned in the paper refers to the generalized cost, not just the economic cost. The effectiveness of each alternative will be evaluated separately in each round of decision-making. Generally, the rigorous alternative can achieve better results, while it may also cause a lot of losses; thus, it is not necessarily the most appropriate alternative. Based on these considerations, the paper proposes the definition and calculation method of the effect per cost, when the predetermined goal can be achieved, we believe that the most appropriate alternative must be the one that has the lowest cost. The establishment of the above theory is also one of the innovations of this paper.

We have to point out some limitations of the paper. As one of the initial conditions, the estimated cost is essentially a fuzzy value, which is difficult to be accurately described by a simple value. Thus, the problem discussed in the paper is actually a double fuzzy problem and more fuzzy variables need to be considered. Further researches will be conducted by our team for this problem in the near future.

## Figures and Tables

**Table 1:** The current severity of the emergency at the initial time point.

Experts	*E*_1_	*E*_2_	⋯	*E*_*m*_
Measured values	*τ*_0_^1^={*γ*_*l*_0_^1^_*|p*_*l*_0_^1^_}	*τ*_0_^2^={*γ*_*l*_0_^2^_*|p*_*l*_0_^2^_}	⋯	*τ*_0_^*m*^={*γ*_*l*_0_^*m*^_*|p*_*l*_0_^*m*^_}

**Table 2:** The estimated severities at the time point *T*_1_.

Experts estimated severities	*E*_1_	*E*_2_	⋯	*E*_*m*_
*τ*_1_^1/′^	*τ*_11_^1/′^	*τ*_12_^1/′^	⋯	*τ*_1*m*_^1/′^
*τ*_1_^2/′^	*τ*_11_^2/′^	*τ*_12_^2/′^	⋯	*τ*_1*m*_^2/′^
⋮	⋮	⋮	⋮	⋮
*τ*_1_^*n*_0_/′^	*τ*_11_^*n*_0_/′^	*τ*_12_^*n*_0_/′^	⋯	*τ*_1*m*_^*n*_0_/′^

**Table 3:** The current severity of the emergency at the first time point.

Experts	*E*_1_	*E*_2_	⋯	*E*_*m*_
Measured values	*τ*_1_^1^={*γ*_*l*_1_^1^_*|p*_*l*_1_^1^_}	*τ*_1_^2^={*γ*_*l*_1_^2^_*|p*_*l*_1_^2^_}	⋯	*τ*_1_^*m*^={*γ*_*l*_1_^*m*^_*|p*_*l*_1_^*m*^_}

**Table 4:** The differences between the estimated values and measured values.

Experts	*E*_1_	*E*_2_	⋯	*E*_*m*_
Differences	*d*_1_^1^=*τ*_11_^*j*/′^ − *τ*_1_^1^	*d*_1_^2^=*τ*_12_^*j*^ ′ − *τ*_1_^2^	⋯	*d*_1_^*m*^=*τ*_1*m*_^*j*/′^ − *τ*_1_^*m*^

**Table 5:** The alternatives proposed by experts at the initial time point.

Alternatives	The specific measures
*A*_0_^1^	We isolate all close contacts and provide disinfection equipment for the dormitories and classrooms visited by close contacts
*A*_0_^2^	In addition to the *A*_0_^1^, we measure the temperature of all the students
*A*_0_^3^	In addition to the *A*_0_^2^, we suspend the courses held by the college of close contacts
*A*_0_^4^	In addition to the *A*_0_^3^, we suspend all the courses and cancel all unnecessary activities among students

**Table 6:** The current severities measured at the initial time point.

Experts	*E*_1_(*ω*_1_=0.3)	*E*_2_(*ω*_2_=0.32)	*E*_3_(*ω*_3_=0.38)
Measured values	(0.1*|*0.4, 0.2*|*0.6)	(0.13*|*0.8, 0.17*|*0.2)	(0.12*|*0.3, 0.17*|*0.2, 0.19*|*0.5)

**Table 7:** The estimated severities at the time point *T*_1_ when using different alternatives.

Experts alternatives	*E*_1_(*ω*_1_=0.3)	*E*_2_(*ω*_2_=0.32)	*E*_3_(*ω*_3_=0.38)
*A*_1_^1^	(0.24*|*0.4, 0.26*|*0.6)	(0.21*|*0.6, 0.23*|*0.3, 0.24*|*0.1)	(0.21*|*0.6, 0.26*|*0.4)
*A*_1_^2^	(0.28*|*0.3, 0.30*|*0.5, 031*|*0.2)	(0.29*|*0.5, 0.31*|*0.5)	(0.29*|*0.3, 0.32*|*0.7)
*A*_1_^3^	(0.36*|*0.3, 0.38*|*0.7)	(0.35*|*0.6, 0.37*|*0.3, 0.39*|*0.1)	(0.33*|*0.5, 0.36*|*0.5)
*A*_1_^4^	(0.38*|*0.3, 0.39*|*0.2, 0.40*|*0.5)	(0.36*|*0.3, 0.40*|*0.7)	(0.38*|*0.6, 0.410.4*|*)

**Table 8:** The current severities measured at the time point *T*_1_.

Experts	*E*_1_(*ω*_1_=0.3)	*E*_2_(*ω*_2_=0.32)	*E*_3_(*ω*_3_=0.38)
Measured values	(0.29*|*0.3, 0.31*|*0.7)	(0.26*|*0.4, 0.3*|*0.6)	(0.32*|*1)

**Table 9:** The differences at the first period.

Experts	*E*_1_(*ω*_1_=0.3)	*E*_2_(*ω*_2_=0.32)	*E*_3_(*ω*_3_=0.38)
Differences	$\left(\begin{array}{c} -0.0|10.09,-0.03|0.21\\ 0.01|0.15,-0.01|0.35\\ 0.02|0.06,0|0.14 \end{array}\right)$	$\left(\begin{array}{c} 0.030.2,-0.01||0.3\\ 0.05|0.2,0.01|0.3 \end{array}\right)$	(−0.03*|*0.3, 0*|*0.7)

**Table 10:** The updated estimated severities of the implemented alternative at the time point *T*_1_.

Experts alternatives	*E*_1_(*ω*_1_=0.3)	*E*_2_(*ω*_2_=0.32)	*E*_3_(*ω*_3_=0.38)
*A*′_0_^2^	(0.28*|*0.3, 0.30*|*0.5, 0.31*|*0.2)	(0.29*|*0.5, 0.31*|*0.5)	$\left(\begin{array}{c} 0.3216081827|0.7\\ 0.29|0.3 \end{array}\right)$

**Table 11:** The updated differences at the first period.

Experts	*E*_1_(*ω*_1_=0.3)	*E*_2_(*ω*_2_=0.32)	*E*_3_(*ω*_3_=0.38)
Differences	$\left(\begin{array}{c} -0.01|0.09,-0.03|0.21\\ 0.01|0.15,-0.01|0.35\\ 0.02|0.06,0|0.14 \end{array}\right)$	$\left(\begin{array}{c} 0.03|0.2,-0.01|0.3\\ 0.05|0.2,0.01|0.3 \end{array}\right)$	$\left(\begin{array}{c} 0.0016081827|0.7\\ -0.03|0.3 \end{array}\right)$

**Table 12:** The estimated severities at the time point *T*_2_ when using different alternatives.

Experts alternatives	*E*_1_(*ω*_1_=0.3)	*E*_2_(*ω*_2_=0.32)	*E*_3_(*ω*_3_=0.38)
*A*_2_^1^	(0.42*|*1)	(0.41*|*0.6, 0.44*|*0.4)	(0.39*|*0.3, 0.43*|*0.7)
*A*_2_^2^	(0.46*|*0.2, 0.5*|*0.8)	(0.47*|*0.5, 0.52*|*0.5)	(0.49*|*0.7, 0.51*|*0.3)
*A*_2_^3^	(0.55*|*0.8, 0.57*|*0.2)	(0.52*|*0.4, 0.56*|*0.6)	(0.54*|*1)
*A*_2_^4^	(0.58*|*0.4, 0.62*|*0.6)	(0.6*|*1)	(0.58*|*0.7*|*0.61*|*0.3)

**Table 13:** The current severities measured at the time point *T*_2_.

Experts	*E*_1_(*ω*_1_=0.3)	*E*_2_(*ω*_2_=0.32)	*E*_3_(*ω*_3_=0.38)
Measured values	(0.63*|*0.5, 0.65*|*0.3, 0.67*|*0.2)	(0.67*|*0.3, 0.68*|*0.7)	(0.66*|*1)

**Table 14:** The differences at the second period.

Experts	*E*_1_(*ω*_1_=0.3)	*E*_2_(*ω*_2_=0.32)	*E*_3_(*ω*_3_=0.38)
Differences	$\left(\begin{array}{c} -0.17|0.10,-0.19|0.06\\ -0.21|0.04,-0.13|0.40\\ -0.15|0.24,-0.17|0.16 \end{array}\right)$	$\left(\begin{array}{c} -0.20|0.15,-0.21|0.35\\ -0.15|0.15,-0.16|0.35 \end{array}\right)$	(−0.17*|*0.7, −0.15*|*0.3)

**Table 15:** The estimated severities at the time point *T*_3_ when using different alternatives.

Experts alternatives	*E*_1_(*ω*_1_=0.3)	*E*_2_(*ω*_2_=0.32)	*E*_3_(*ω*_3_=0.38)
*A*_3_^1^	(0.88*|*0.2, 0.92*|*0.2)	(0.91*|*0.6, 0.93*|*0.4)	(0.82*|*1)
*A*_3_^2^	(0.94*|*0.4, 0.96*|*0.6)	(0.95*|*1)	(0.93*|*0.7, 0.96*|*0.3)
*A*_3_^3^	(0.97*|*0.5, 0.98*|*0.5)	(0.95*|*0.4, 0.97*|*0.6)	(0.96*|*1)
*A*_3_^4^	(0.97*|*1)	(0.98*|*0.4, 0.99*|*0.6)	(0.94*|*0.7*|*0.96*|*0.3)

**Table 16:** The current severities measured at the time point *T*_3_.

Experts	*E*_1_(*ω*_1_=0.3)	*E*_2_(*ω*_2_=0.32)	*E*_3_(*ω*_3_=0.38)
Measured values	(0.95*|*0.4, 0.98*|*0.6)	(0.97*|*1)	(0.96*|*0.8, 0.98*|*0.2)

**Table 17:** The conversion values in the form of hesitant fuzzy sets.

Experts alternatives	*E*_1_(*ω*_1_=0.3)	*E*_2_(*ω*_2_=0.32)	*E*_3_(*ω*_3_=0.38)
*A*_1_^1^	(0.24, 0.26)	(0.21, 0.23, 0.24)	(0.21, 0.26)
*A*_1_^2^	(0.28, 0.30, 0.31)	(0.29, 0.31)	(0.29, 0.32)
*A*_1_^3^	(0.36, 0.38)	(0.35, 0.37, 0.39)	(0.33, 0.36)
*A*_1_^4^	(0.38, 0.39, 0.40)	(0.36, 0.40)	(0.38, 0.41)

**Table 18:** The transformed values in the form of the probabilistic hesitant fuzzy sets.

Experts alternatives	*E*_1_(*ω*_1_=0.3)	*E*_2_(*ω*_2_=0.32)	*E*_3_(*ω*_3_=0.38)
*A*_1_^1^	(*s*_1_(1))	(*s*_1_(1))	(*s*_1_(1))
*A*_1_^2^	(*s*_1_(1))	(*s*_1_(1))	(*s*_1_(1))
*A*_1_^3^	(*s*_1_(1))	(*s*_1_(1))	(*s*_1_(1))
*A*_1_^4^	(*s*_1_(0.5), *s*_2_(0.5))	(*s*_1_(0.3), *s*_2_(0.7))	(*s*_1_(0.6), *s*_2_(0.3))

## Data Availability

The data used to support the findings of this study are included within the article, and they are obtained through practical investigations.
